# Alpha adrenergic modulation on effects of norepinephrine transporter inhibitor reboxetine in five-choice serial reaction time task

**DOI:** 10.1186/1423-0127-16-72

**Published:** 2009-08-14

**Authors:** Yia-Ping Liu, Yu-Lung Lin, Chia-Hsin Chuang, Yu-Cheng Kao, Shang-Tang Chang, Che-Se Tung

**Affiliations:** 1 Dept of Physiology and Biophysics, National Defense Medical Center, Taipei, Taiwan, Republic of China; 2 Dept of Medical Research Center, Cardinal Tien Hospital, Taipei, Taiwan, Republic of China; 3 Dept of Psychiatry, Song-Shan Armed Forces General Hospital, Taipei, Taiwan, Republic of China; 4 Dept of Psychiatry, Cardinal Tien Hospital, Taipei, Taiwan, Republic of China

## Abstract

The study examined the effects of a norepinephrine transporter (NET) inhibitor reboxetine (RBX) on an attentional performance test. Adult SD rats trained with five-choice serial reaction time task (5-CSRTT) were administered with RBX (0, 3.0 and 10 mg/kg) in the testing day. Alpha-1 adrenergic receptor antagonist PRA and alpha-2 adrenergic receptor antagonist RX821002 were used to clarify the RBX effect. Results revealed that rat received RBX at 10 mg/kg had an increase in the percentage of the correct response and decreases in the numbers of premature response. Alpha-1 adrenergic receptor antagonist Prazosin (PRA) at 0.1 mg/kg reversed the RBX augmented correct responding rate. However, alpha-2 adrenergic receptor antagonist RX821002 at 0.05 and 0.1 mg/kg dose dependently reversed the RBX reduced impulsive responding. Our results suggested that RBX as a norepinephrine transporter inhibitor can be beneficial in both attentional accuracy and response control and alpha-1 and alpha-2 adrenergic receptors might be involved differently.

## Background

The action of norepinephrine (NE) can be terminated not only by enzymes that destroy NE but also by a transport pump for NE, i.e., removing it from the synapse without destroying it. The transport pump that terminates the synaptic action of NE is called NE "transporter" or abbreviated as NET. NET locates in the pre-synaptic terminal and acts to remove NE out of the synaptic cleft thus stops its action [1]. In terms many issues of the selectivity and mechanisms of action remained unresolved, selective NET inhibitor has now been developed to treat a variety of brain-related disorders, including depression, attention deficit hyperactivity disorder (ADHD), post-traumatic stress disorder (PTSD) and cocaine dependence [1-4]. Reboxetine (RBX) is the first potent, selective and specific NE transporter inhibitor that has been marketed as an antidepressant [5]. Most of the previous studies focused on the antidepressant activity of RBX in rodents [6] in which RBX exhibited an exceptional antidepressant effect [7]. Nevertheless there is relatively lack of study examining the effect of RBX on the aspect of response control and attentional function. The latter is worth to address given the fact the disturbed amount of synaptic NE generally associated with the symptoms such as impaired attention, problems concentrating, slowness of information processing and poor response control [8,9]. The present experiment therefore aimed to examine the RBX-induced attentional changes by employing a recognized behavioral test, named five-choice serial reaction time task (5-CSRTT).

The 5-CSRTT was modeled after Leonard's five-choice serial task [10], which was commonly used to assess the behavioral effects of different forms of arousal in humans [11]. In the 5-CSRTT, animals are required to discriminate, spatially, a short visual stimulus occurring randomly in one of five locations after a scheduled waiting period. The test requires that the rat must be able to attend to the array of openings in a specially designed apparatus in order to detect the discriminative stimulus and respond correctly to it. Accurate responding requires attention both in the temporal and spatial domains, thus, providing a high degree of parametric flexibility and the potential for independent assessments of the spatial and temporal components of attention [12]. Furthermore, the 5-CSRTT provides measures of 'premature' responding, as the animals need to wait for the imperative visual stimulus prior to making a discriminative response [13]. The 5-CSRTT could be widely used to separate animals with deficits in their attentional processes from other behavioral or cognitive features, for example, locomotor activity when adding with a pair of infra-red beams in the testing box, food-related motivation in terms of the speed in collecting earned food pellets [14] and motoric impulsivity as recording the numbers of premature response prior to the occurrence of visual signal [15,16].

In the present study, the 5-CSRTT was aimed to manifest drug-induced attention/impulsivity modifications by clarifying whether RBX treated rats could exhibit less premature responding or better accuracy of detecting visual target stimuli as they did when applied to humans in the recovery from depressive state. The authors designed a series of experiments to investigate the effects of RBX on the performance of the 5-CSRTT in rats in order to assess the benefits of RBX concerning the function of visual attention and the capacity of response control. The performance of 5-CSRTT was examined in rats following the administration of RBX, PRA and RX821002. The latter two were selective agents to antagonize alpha-1 and alpha-2 adrenergic receptors, respectively, and thus were used to test the possibility of reversing the RBX effects on the performance of the 5-CSRTT. The results obtained from this study may contribute to the understanding of the functional role of NET inhibitor and be helpful for further clinical implementations.

## Materials and methods

### Subjects

A total of 57 male Sprague-Dawley rats (National Laboratory Animal Center, Taiwan R.O.C.) aged approximately 4 months and weighted 300-350 gm at the start of the experiment were selected. All rats were housed in a temperature-humidity-controlled holding facility with 12-hour light/dark cycles (light on from 07:00 to 19:00). All rates received ad libitum water. However, food was restricted to that earned during the test sessions [maximum of 100 × 45 mg purified rodent pellets (Labdiet, USA) and 20 gm/rat standard rodent chow (TSE, Taiwan) at the end of the test. The test took place between 08:00 and 18:00 with all rats being tested at the same time every day where possible. All experiment procedures were evaluated and permitted to perform by the animal care committee of National Defense Medical Center with the permission code number IACUC-04-156. All efforts were made to reduce the numbers of animals used and to minimize animal suffering during the experiment.

### Apparatus

The apparatus (TSE, Germany) used in this study was very similar to those described in other studies [17,18]. It consisted of four 25 × 25 cm aluminium operant chambers without levers and each of them accompanied with a curved rear wall. Set into the curved wall were five, 2.5 cm square holes, 4 cm deep and 2.5 cm above a wire mesh floor. Each hole had an infrared photocell beam to monitor its entrance. Illumination of each hole was provided by a 3W bulb located at the rear of the hole. Reinforcement was provided by a food-pellet dispenser to deliver pellets into a magazine at the front of the chamber. The chamber was illuminated by a 3W house-light mounted in the centre of the ceiling. The rats were introduced to the chamber through a hinged Perspex flap in the top half of the front wall. The chamber was contained within a sound-attenuating wooden box. Ventilation was supplied by means of a fan, providing a low level of background noise that masked extraneous sound. On-line control of the apparatus and data collection were performed by using a microcomputer.

### Training procedure

#### Food deprivation

Rats were instructed to the restricted feeding regimen one week prior to training programs with standard rodent chow, and gradually reached 90% of their free feeding weight.

#### Magazine training

Initial shaping consisted of a magazine training, whereby the rats learned that food was available in the magazine in which the hinged metal panel was wedged open with surplus pellets. At this stage, rats were exposed to the boxes for 15 minutes per session. Once all rats reliably ate all food pellets within a session, the rats were moved onto a fixed interval schedule.

#### Fixed interval schedule

From the following 3 days, rats were placed in the dark chamber 20 minutes per day with a fixed time schedule, in which a food pellet was delivered every 20 seconds into the magazine.

#### Five-choice serial reaction time task (5-CSRTT)

**Figure 1:**
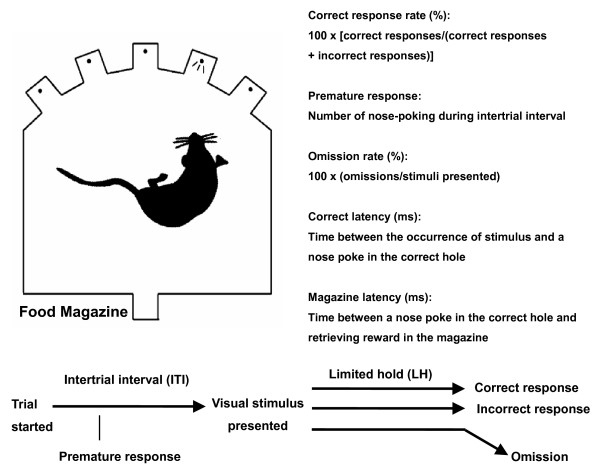
**Schematic illustration of the 5-CSRTT used in the present experiment**. Rat was placed in the center of the chamber. A trial started with the illumination of magazine until rat made a nose-poke response. After a delay (the intertrial interval, ITI), the stimulus light was presented over the hole in pseudorandom order. If an animal respond with a nose-poke into the illuminated hole within a limited hold of 5 s, a pellet was delivered into the magazine. An ITI began when the food pellet was retrieved, with the next stimulus light presented after the ITI. A period of time-out would be followed by an incorrect response with the house-light on for 5 seconds, during which no stimuli were presented. After a timeout, the rat could start a new ITI by nose-poking to magazine. Analyses of measures were based on the formulations as indexed.

Every rat was consistently placed in the same chamber and well-trained in the 5-CSRTT. A session terminated after a maximum of 100 completed trials or after 30 minutes. A trial started with the illumination of magazine until rats to response with a nose-poke response. After a delay (the intertrial interval, ITI), the stimulus light was presented over the hole in pseudorandom order. If the rat then broke a photo-beam crossing by nose-poke within a limited hold of 5 s, a food pellet was delivered into the magazine. An ITI began if the food pellet was retrieved and the next stimulus light presented after the ITI. A period of time-out (i.e., a period of darkness for 5 seconds) would be followed by an incorrect response or an error of omission with the house-light extinguished, and during which no stimuli were presented. After a time-out, the rats started a new ITI by nose poking to magazine. Figure 1 provided a schematic illustration of 5-CSRTT used in the present study.

The acquisition of the 5-CSRTT was divided into several sub-stages based on a scheduled stepped descending sequence of stimulus duration at 60, 32, 16, 8, 4, 2, 1.5 and finally 1.0 seconds. For these sub-stages, rats were required to meet all of the following criteria in continuous 3 sessions before passing on to the next training stage: (1) More than 80 trials were carried out within a period of 30 minutes; (2) Omission rate was less than 20%; (3) An accuracy rate of 80% or more was achieved; and (4) The numbers of premature response were less than 30.

### Measures and analysis

Accuracy of performance was measured by the calculation of the percentage of correct response [correct responses/(correct responses + incorrect responses)]. An error of omission was scored if the rat failed to respond sufficiently soon after the stimulus onset. A premature response was scored if the rat responded in any hole during the ITI. To assess speed of responding, correct latency was measured as time elapsing between the occurrence of visual stimulus and a nose poke in the correct hole. Magazine latency was measured as time elapsing between a nose poke in the correct hole and retrieving reward in the magazine.

Obtained data was analyzed using SPSS (version 12.0) for variables of interest. In experiment 1, one-way ANOVA was conducted with dose as a non-repeated factor. In experiment 2, one-way ANOVA was conducted with dose as a repeated factor. In experiment 3, two-way ANOVA was conducted with both treatment and dose as repeated factors. Further analyses and post-hoc comparisons were performed if necessary. A *p *value < 0.05 was considered statistically significant.

### Drugs and experimental design

#### Drugs

The performance of 5-CSRTT was examined after the administration of vehicle (0.9% saline solution) or the following drugs: RBX (sponsored by Pfizer Pharmaceutics, USA), PRA (Sigma, USA) and RX821002 (Sigma, USA). All drugs were administered in a volume of 2 ml/kg and were diluted in isotonic saline. RBX was administered intraperitoneally 60 minutes prior to the test whereas for PRA or RX8212002, 10 minutes before the test. The doses used were 10 mg/kg for RBX, 0.1 and 0.3 mg/kg for PRA, and 0.05 and 0.1 mg/kg for RX821002.

#### Experiment 1: dose-response study of RBX in the 5-CSRTT

Twenty-four rats reached the acquisition criteria level of 5-CSRTT (i.e., 1.0 sec of stimulus duration) were used. Rats were tested their 5-CSRTT performance after the administration of RBX (0, 3 and 10 mg/kg). The number of animal was 8 for each dose. RBX was injected intraperitoneally 60 minutes prior to the test.

#### Experiment 2: dose-response study of PRA and RX821002 in the 5CSRTT

Twenty-three rats were examined their 5-CSRTT performance 10 min later following PRA (N = 11) or RX821002 (N = 12) administration. Each rat received three dose conditions (for PRA, 0, 0.1 and 0.3 mg/kg; for RX821002, 0, 0.05 and 0.1 mg/kg). The order of injection condition was based on a Latin Square design. Each test session was preceded by two control sessions in which saline vehicle was administered.

#### Experiment 3: The interaction of RBX with PRA or RX in the 5-CSRTT

Ten rats were examined their 5-CSRTT performance under the following 4 conditions (vehicle + vehicle, RBX + vehicle, vehicle + PRA, RBX + PRA). The dose for RBX was 10 mg/kg and for PRA, 0.3 mg/kg. Another 10 rats were examined their 5-CSRTT performance under the following 4 conditions (vehicle + vehicle, RBX + vehicle, vehicle + RX821002, RBX + RX821002). The dose for RBX was 10 mg/kg and for RX821002, 0.1 mg/kg. The order of injection condition was based on a Latin Square design. Each test session was preceded by two control sessions in which saline vehicle was administered.

## Results

### Effect of RBX on 5-CSRTT

**Figure 2:**
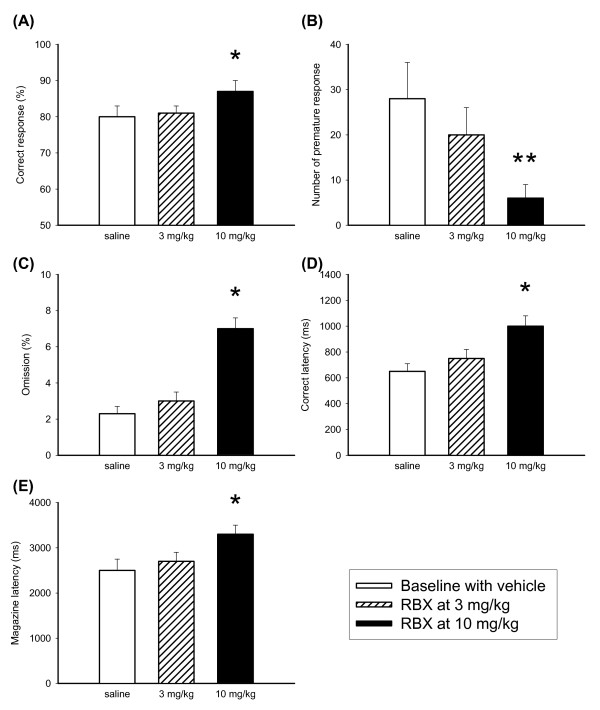
**Effects of RBX on the performance of 5-CSRTT**. The graphs showed the effects of RBX (0, 3.0 and 10 mg/kg, N = 8 for each dose) on the performance of 5-CSRTT in the aspects of correct response rate (A), premature response (B), omission rate (C), correct latency (D) and magazine latency (E). Open bars depicted baseline vehicle controls, oblique lattice bars depicted RBX at 3 mg/kg, and black bars depicted RBX at 10 mg/kg. Values are presented as mean+SEM. Student-t test were used for each RBX treatment as compared with vehicle condition, * *P *< 0.05, ** *P *< 0.01.

Effects of RBX on performance of the 5-CSRTT were shown in Figure 2. RBX was found to altered rats' 5-CSRTT performance in all aspects. (correct response, F(2,21) = 3.89, p < 0.05; premature response, F(2,21) = 6.33, p < 0.01; omission rate, F(2,21) = 4.24, p < 0.05; correct latency, F(2,21) = 6.86, p < 0.01 and magazine latency, F(2,21)= 7.01, p < 0.01). Further analyses by Dunnett's test revealed that the difference was mainly contributed from the condition between vehicle and 10 mg/kg RBX.

### Effects of PRA or RX821002 on 5-CSRTT

**Figure 3:**
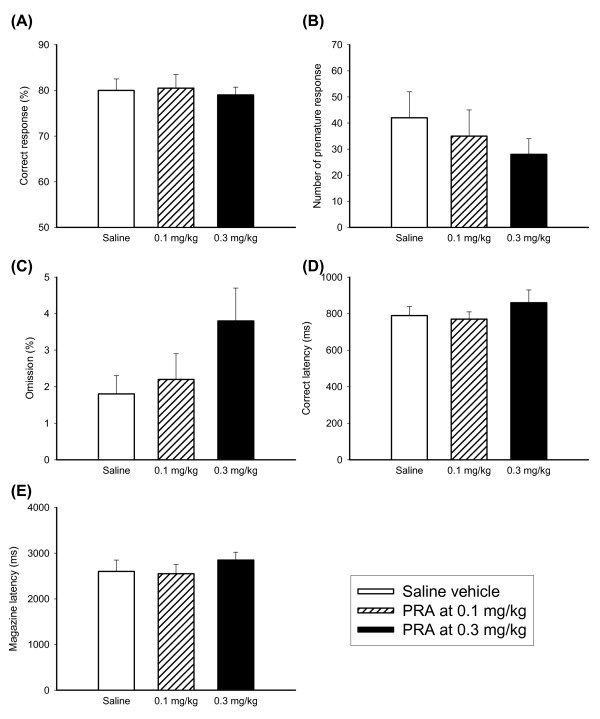
**Effects of PRA on the performance of 5-CSRTT**. The graphs showed the effects of different dosage of PRA (0, 0.1 and 0.3 mg/kg) on the performance of 5-CSRTT (N = 11) in the aspects of correct response rate (A), premature response (B), omission rate (C), correct latency (D) and magazine latency (E). Open bars depicted saline vehicle controls, oblique lattice bars depicted PRA at 0.1 mg/kg, and black bars depicted PRA at 0.3 mg/kg. Values are presented as mean+SEM. * *P *< 0.05.

**Figure 4:**
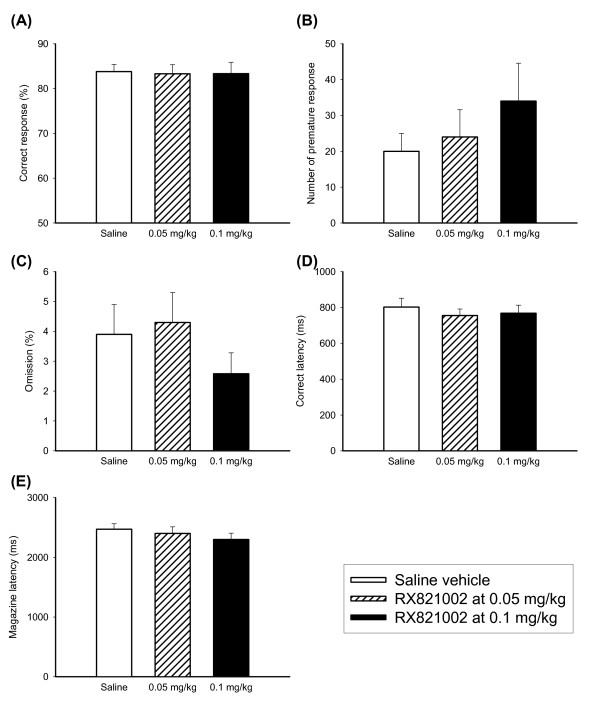
**Effects of RX821002 on the performance of 5-CSRTT**. The graphs showed the effects of different dosage of RX821002 (0, 0.05 and 0.1 mg/kg) on the performance of 5-CSRTT (N = 12) in the aspects of correct response rate (A), premature response (B), omission rate (C), correct latency (D) and magazine latency (E). Open bars depicted saline vehicle controls, oblique lattice bars depicted RX821002 at 0.05 mg/kg, and black bars depicted RX821002 at 0.1 mg/kg. Values are presented as mean+SEM. * *P *< 0.05.

Effects of alpha-1 adrenergic receptor antagonist PRA were shown in Figure 3. PRA appeared to have no effect on any of the measures [F(2,20) = 0.18 for correct response; F(2,20) = 0.72 for premature response; F(2,20) = 0.78 for omission rate; F(2,20) = 0.31 for correct latency and F(2,20) = 0.48 for magazine latency]. Effects of alpha-2 adrenergic receptor antagonist RX821002 were shown in Figure 4. RX821002 had minor effects on accurate responding and latency to respond or collect food [F(2,22) = 0.02 for correct response; F(2,22) = 0.66 for premature response; F(2,22) = 0.65 for omission rate; F(2,22) = 0.40 for correct latency and F(2,22) = 0.58 for magazine latency].

### Effects of RBX on 5-CSRTT following pretreatment with PRA

**Figure 5:**
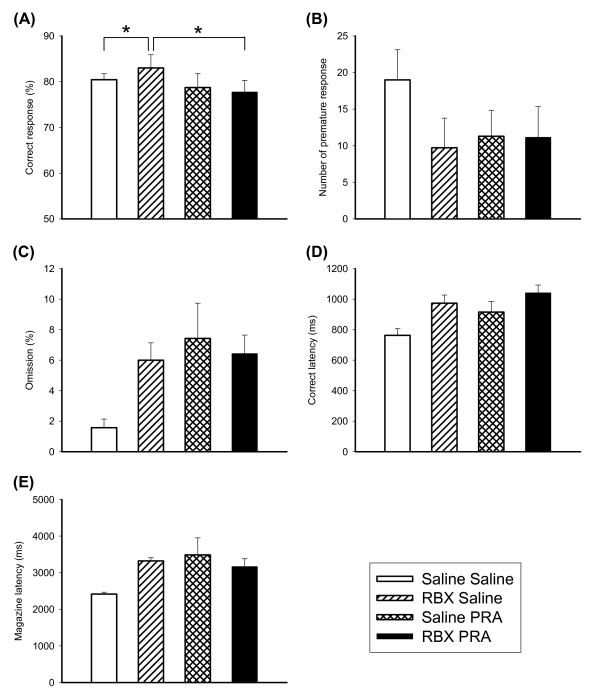
**Effects of RBX in combination with PRA on the performance of 5-CSRTT**. The graphs showed the effects of RBX 10 mg/kg in combination with PRA 0.3 mg/kg (N = 10) on the performance of 5-CSRTT in the aspects of correct response (A), premature response (B), omission rate (C), correct latency (D) and magazine latency (E). Open bars depicted saline + saline, oblique lattice bars depicted RBX + saline, reticular bars depicted saline + PRA, and black bars depicted RBX + PRA. Values are presented as mean+SEM. * P < 0.05.

Effects of RBX on performance of the 5-CSRTT in the condition of pretreatment of alpha-1 adrenergic receptor antagonist PRA (0.3 mg/kg) were examined (see Figure 5). There was a significant effect of RBX × PRA in correct response [F(1,9) = 6.47, p < 0.05]. Simple main effect was found in the condition of RBX without PRA [F(1,9) = 5.43, p < 0.05]. Further comparisons revealed there were differences between RBX-saline and saline-saline [t(1,9) = 2.34, p < 0.05], and RBX-saline and RBX-PRA [t(1,9) = 2.66, p < 0.05]. For premature response, main effects were found in RBX [F(1,9) = 10.98, p < 0.01] and PRA [F(1,9) = 7.11, p < 0.05] without interactions. For omission rate, main effects were found in RBX [F(1,9) = 11.42, p < 0.01] and PRA [F(1,9) = 11.61, p < 0.01] without interactions. For correct latency and magazine latency, main effect of RBX was found significant [F(1,9) = 9.05, p < 0.05 and F(1,9) = 11.78, p < 0.01].

### Effects of RBX on 5-CSRTT following pretreatment with RX821002

**Figure 6:**
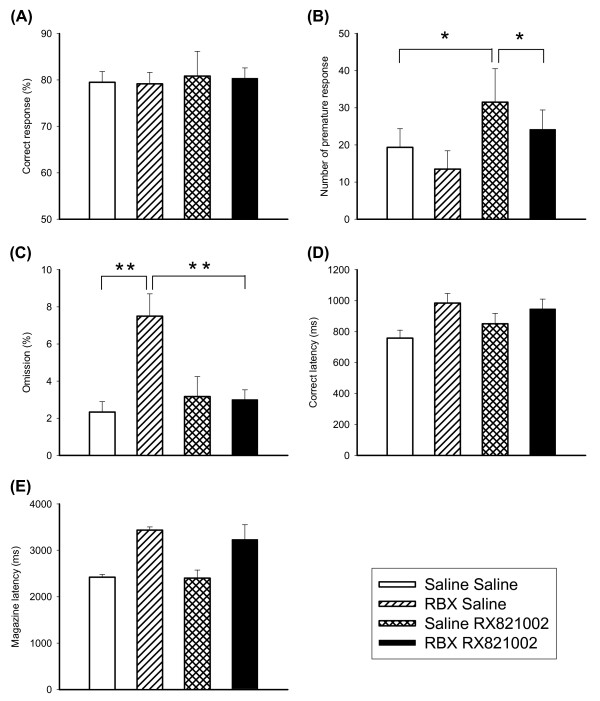
**Effects of RBX in combination with RX821002 on the performance of 5-CSRTT**. The graphs showed the effects of RBX 10 mg/kg in combination with RX821002 0.1 mg/kg (N = 10) on the performance of 5-CSRTT in the aspects of correct response rate (A), premature response (B), omission rate (C), correct latency (D) and magazine latency (E). Open bars depicted saline + saline, oblique lattice bars depicted RBX + saline, reticular bars depicted saline + RX821002, and black bars depicted RBX + RX821002. Values are presented as mean+SEM. * P < 0.05.

Effects of RBX on performance of the 5-CSRTT in the condition of pretreatment of alpha-2 adrenergic receptor antagonist RX821002 (0.1 mg/kg) were examined (see Figure 6). In correct response, there was little effect on RBX, RX821002 or RBX × RX821002. In premature response, There was a significant effect of RBX × RX821002 [F(1,9) = 6.08, p < 0.05]. Simple main effect was found in the condition of RX821002 without RBX [F(1,9) = 5.77, p < 0.05]. Further comparisons revealed there were differences between saline-saline and saline-RX821002 [t(1,9) = 2.72, p < 0.05), and saline-RX821002 and RBX-RX821002 (t(1,9) = 4.11, p < 0.01). For omission rate, There was a significant effect of RBX × RX821002 [F(1,9) = 11.62, p < 0.01]. Simple main effect was found in the condition of RBX without RX821002 [F(1,9) = 5.85, p < 0.05]. Further comparisons revealed there were differences between saline-saline and RBX-saline [t(1,9) = 6.56, p < 0.001], and RBX-saline and RBX-RX821002 [t(1,9) = 5.93, p < 0.001]. For correct latency and magazine latency, main effect of RBX was found significant [F(1,9) = 8.77, p < 0.05 and F(1,9) = 6.30, p < 0.05].

## Discussion

The present study described NET inhibitor RBX could improve attentional function and the ability of response control in a task requiring visual accuracy and the management of impulsiveness. The contributions of alpha-1 and alpha-2 adrenoceptors in the RBX effect were examined. While the alpha-1 effect was mainly responsible for attentional accuracy, Alpha-2 adrenergic receptors contributed to the adjustment of impulsiveness. Interpretations of these findings were discussed as the following.

For protocol used in the present study, behavior was mainly controlled by the rats' attentional function and their motor impulsiveness. For example, rats made their correct response largely depending on their sustained and divided attentional functions. At the same time, rats also had to restrain their impetuous nature for not nose-poking hastily, which was representative of their ability to control motor impulsiveness. Attentional impairment has been considered for a long time as one of the major cognitive psychopathologies of major depression [19-21]. The beneficial effect of NET inhibitor reboxetine found in this study was in line with the concept that the attentional and executive disturbance in major depressive patients could be alleviated following the treatment of antidepressant medications [22].

In general it takes al least two weeks for antidepressants to attain their clinical efficacy. Accordingly in the present study, we demonstrated RBX treatment for unremittingly two weeks appeared to produce different effects on attention and impulsiveness. As their impulsiveness remained unaffected, rats under RBX treatment became less attentive. Attentional performance and impulse control had been assumed based on distinctive neurobiological mechanisms and they both depended largely on the integrity of prefrontal cortex, particularly dorsa-lateral prefrontal cortex. The present experiment demonstrated that when RBX was combined with alpha-2 antagonist RX821002, the latter successfully reversed the RBX improved impulse control in 5-CSRTT. The result supported the idea that when analyzing the improvement of rats' attentional performance, selection response and impulse control might be involved in a dissociated manner [18]. The present study continued to extend the idea and found alpha-1 and alpha-2 adrenoceptors might contribute in a different way to accuracy and vigor responding.

Central noradrenergic system was considered significantly being involved in the regulation of attentional function. For the knowledge to date, alpha-1 adrenoceptor revealed a greater extent than alpha-2 adrenoceptor in modulating response accuracy in the task concentrating subject's attention [23]. Systemic administration of alpha-1 agonist St-587 improved the response accuracy and this effect could be blocked by alpha-1 receptor antagonist prozasin [24]. Activation of alpha-1 adrenergic receptor manifested importantly in conditions requiring attention. For example, alpha-1 adrenergic activation facilitated cognitive performance of rats in situation requiring a variety of arousal levels, such as attentional set-shifting task [25]. The present study was in the other way to demonstrate that the alpha-1 adrenergic receptor was crucial for both sustained and divided attentional functions [23,24,26,27]. Apparently in 5-CSRTT of the present study, the action of motor impulsiveness occurred prior to the emergency of any corresponding visual signal, therefore it inherited with anticipatory character. Alpha-1 antagonist PRA when given alone had a minor effect on the anticipatory response in 5-CSRTT. Previously PRA at the dose of 0.1 mg/kg was found unable to reverse the effect of alpha-1 agonist St-587 in reducing premature responses [24]. This was consistent with what happened in the present experiment as PRA at the same dose could not abolish the RBX induced reduction of premature response.

Taken together it appeared that the alpha-1 adrenoceptor contributed little in controlling anticipatory actions in situation requiring sustained and divided attentions. On the other hand, the role of alpha-2 adrenoceptor in the regulations of motor impulse control might be more important. Our results demonstrated that although alpha-2 adrenoceptor antagonist per se was little to do with premature responding as revealed before [4], it may potentially help to adjust the effect of NET inhibitor. Possible mechanisms might be related to either an increase of norepinephrine availability following presynaptic auto-regulation [28], or the postsynaptic mechanism through which the alpha-2 adrenoceptor antagonist abolished the subsequent effect following RBX induced norepinephrine surge. The former seemed less plausible due to the following considerations. First, the effects of an alpha 2-adrenoceptor agonist dexmedetomidine on anticipatory responses were found no difference between controls and rats with central NA depleted by N-(2-chloroethyl)-N-ethyl-2-bromobenzy-lamine (DSP-4). Second, alpha-2 adrenoceptor may function as a terminal presynaptic autoreceptor to restrain NE release [29]; or heteroreceptor at 5HT terminal to hold back 5HT release [29,30]. In the present study if the enhanced ability of impulse control by RBX was attributable to the acceleration of 5HT neuronal activity via alpha-1 adrenoceptor at 5HT cell body, the activation of alpha-2 autoreceptor located on NA terminal on the other hand would hold back the release of NE, that in turn attenuated alpha-1 activation therefore rats should be more impulsive.

In attentional task as 5-CSRTT, measures in fact provided valuable information other than visual accuracy and impulse control. For example, correct latency generally refers to the reaction time elapsed from coming out visual signal to the rats' nose-poking the correct hole. Increase of correct latency suggests a longer motor readiness or hypoactivity [31]. Compared to correct latency, magazine latency refers to the reaction time elapsed from coming out the food pallet to the collection of that reward, normally reflecting animals' motivational state. In the present experiment, correct latency was found longer in both treatment conditions, RBX alone and RBX combined with PRA or RX821002. Magazine latency was also longer following RBX treatment. The similar impacts over correct response time and food collecting time suggested the reaction time was prolonged, no matter the target stimulus was reward itself or its antecedent. However, the cognitive benefit of RBX in reducing the premature response was unlikely caused by the prolonged reaction time since the elapse duration of correct reaction time was around 200 ms (i.e., 0.2 second), far from the time scale of impulsiveness indexed by the premature response counted within the scale of 5 sec ITI.

Along with many other antidepressants, the peripheral effect of RBX is well worth considering in which autonomic side effects such as dry mouth and adjusted sympathetic vasomotor tone had been reported previously [32,33]. This might elicit the concerning that the result obtained form the present experiment might be contributed to a degree by peripheral effects of RBX. Although this issue was worth taking into account; it was not much likely to confound the interpretation of the result. In an experimental design such as 5CSRTT, it was the level of motor function more responsible for influencing the result of 5-CSRTT. In the present study, animals' motor function was preserved well since both correct and magazine latencies did not prolong following the administration of RBX.

In summary, the present study demonstrated NET inhibitor RBX may exhibit some potential benefit in the aspects of both response accuracy and behavioral inhibition. Future studies would suggest validating the argument obtained from the present study in a more comprehensive way, for example, examining the effect of RBX on signal transduction pathways mediated by alpha-1 and -2 adrenergic receptors.

## Competing interests

The authors declare that they have no competing interests.

## Authors' contributions

YPL and YLL carried out the 5-CSRTT work. CHC was responsible for drug administration. YCK and STC were responsible for clinical interpretation. YPL and CST conceived of the study, and participated in its design and coordination. All authors read and approved the final manuscript.
